# ProHealth eCoach: user-centered design and development of an eCoach app to promote healthy lifestyle with personalized activity recommendations

**DOI:** 10.1186/s12913-022-08441-0

**Published:** 2022-09-04

**Authors:** Ayan Chatterjee, Andreas Prinz, Martin Gerdes, Santiago Martinez, Nibedita Pahari, Yogesh Kumar Meena

**Affiliations:** 1 Department of Information and Communication Technology, Centre for e-Health, University of Agder, Jon Lilletuns Vei 9, Grimstad, Norway; 2 Department of Health and Nursing Science, Centre for e-Health, University of Agder, Grimstad, Norway; 3 Department of Software Development, KnowIT AS, Kristiansand, Norway; 4 School of Computer Science and Electronic Engineering, University of Essex, Colchester, UK

**Keywords:** eCoach, User-centered Design, Persuasive strategies, Visualization, Healthy lifestyle, Physical activity

## Abstract

**Background:**

Regular physical activity (PA), healthy habits, and an appropriate diet are recommended guidelines to maintain a healthy lifestyle. A healthy lifestyle can help to avoid chronic diseases and long-term illnesses. A monitoring and automatic personalized lifestyle recommendation system (i.e., automatic electronic coach or eCoach) with considering clinical and ethical guidelines, individual health status, condition, and preferences may successfully help participants to follow recommendations to maintain a healthy lifestyle. As a prerequisite for the prototype design of such a helpful eCoach system, it is essential to involve the end-users and subject-matter experts throughout the iterative design process.

**Methods:**

We used an iterative user-centered design (UCD) approach to understend context of use and to collect qualitative data to develop a roadmap for self-management with eCoaching. We involved researchers, non-technical and technical, health professionals, subject-matter experts, and potential end-users in design process. We designed and developed the eCoach prototype in two stages, adopting different phases of the iterative design process. In design workshop 1, we focused on identifying end-users, understanding the user’s context, specifying user requirements, designing and developing an initial low-fidelity eCoach prototype. In design workshop 2, we focused on maturing the low-fidelity solution design and development for the visualization of continuous and discrete data, artificial intelligence (AI)-based interval forecasting, personalized recommendations, and activity goals.

**Results:**

The iterative design process helped to develop a working prototype of eCoach system that meets end-user’s requirements and expectations towards an effective recommendation visualization, considering diversity in culture, quality of life, and human values. The design provides an early version of the solution, consisting of wearable technology, a mobile app following the “Google Material Design” guidelines, and web content for self-monitoring, goal setting, and lifestyle recommendations in an engaging manner between the eCoach app and end-users.

**Conclusions:**

The adopted iterative design process brings in a design focus on the user and their needs at each phase. Throughout the design process, users have been involved at the heart of the design to create a working research prototype to improve the fit between technology, end-user, and researchers. Furthermore, we performed a technological readiness study of ProHealth eCoach against standard levels set by European Union (EU).

**Supplementary Information:**

The online version contains supplementary material available at 10.1186/s12913-022-08441-0.

## Key contributions to the literature

- This study proposes ProHealth eCoach to promote a healthy lifestyle with personalized activity recommendations using iterative design process.
- The foremost principle of this eCoach system is to reinforce positive behavior by persuasive strategies, such as self-monitoring of behavior, self-management, personalization, goal setting, reminder, rewards, personalized recommendation generation, and its effective presentation.
- Based on insights from a series of design workshops, we envisage PA as the basis for a healthy lifestyle and the development of the eCoach system. However, regardless of the design and development of multiple PA apps in the Appstore and Playstore, it remains unclear how to design and develop an engaging and effective PA coaching app.

## Background

Chronic illness associated with modifiable lifestyle factors will be accountable for the highest death rates worldwide [1–7]. Lack of physical activities, improper dietary habits, excess consumption of tobacco and alcohol are severe risk factors for chronic diseases, such as obesity, overweight, hypertension, diabetes type II, cardiovascular diseases (CVDs), osteoporosis, and several types of cancer [1–7]. The World Health Organization (WHO) recommends for adults aged 18–64 years at least 150–300 minutes of moderate-intensity aerobic exercise or at least 75–150 minutes of high-intensity aerobic exercise or an equivalent combination of medium and high-intensity exercise throughout the week [6, 7]. Furthermore, according to WHO, adults should include at least 400 g or five servings daily of fruits, vegetables, legumes (i.e., lentils and legumes, nuts, and whole grains (i.e., unprocessed corn, millet, oats, wheat, and brown rice) in their healthy dietary plan [8]. Previous studies have shown that an active and healthy lifestyle can reduce the risk of chronic disease and improve the health-related quality of life and psychological condition of people suffering from chronic illness [1–7]. Healthy lifestyle management can be supported by self-management, motivation, coaching, regular monitoring, goal setting, goal evaluation reminders, and contextual personalized recommendation generation. Persuasive approaches such as eCoaching can empower people to manage a healthy lifestyle with early risk predictions and appropriate individualized recommendations. The intended eCoach system is a set of computerized components that constitutes an artificial entity that can observe, reason about, learn from and predict a user’s behaviors in context and over time, and that engages proactively in an ongoing collaborative conversation with the user to aid planning and to promote practical goal striving with persuasive techniques [9].

### mHealth interventions and factors for physical activity behavior

There exist multiple challenges in developing a mHealth mobile app, such as involvement of different stakeholders, consideration of needs and preferences of end-users from diverse backgrounds, time, technical limitations, and practical implementation of behavioral coaching theories in the app design [10]. To develop an effective mHealth intervention, the following popular frameworks have been suggested – Integrate, Design, Assess, and Share framework (IDEAS), Medical Research Council (MRC) framework, behavioral intervention technology (BIT) model [10]. Moreover, interventions should identify target behavior and factors responsible for behavior change [10]. The notable PA behavior factors are - goals, motivation, habits, emotions, perceived risks, and contextual influences [10]. The challenge in developing a mHealth mobile app lies in applying PA behavior factors. Literature shows different existing models [10], such as capability, opportunity, motivation, and behavior (COM-B) model, self-determination theory, socio-ecological models, goal-getting theory, behavior economics, Fogg’s behavior model (FBM), and just-in-time (adaptive) interventions (JITAIs). Sporrel et al. [10] expected that the individual ability and motivation have a high chance of engaging in physical activity, and if the participant receives personalized recommendations at such a moment, s/he will participate in the physical activity with enthusiasm.

### Aim of the study

The design and development of a health eCoach system require integration between technologies (e.g., mobile phone, computer, wearable and non-wearable sensors, tablet), concepts and strategies from interdisciplinary domains (health informatics, computer science, software engineering, persuasive technologies, networking, and human-computer-interaction (HCI)), and of users’ preferences and requirements in an engaging manner. The UCD approach [11–15] may solve such an integration challenge by positioning the end-users centrally for designing, developing, testing, and evaluating an eCoach prototype. It may promote interactive digital services and applications with Internet-of-Things (IoT) connected sensors and actuators to open new opportunities for HCI [16, 17]. A user-centered design framework integrates a wide range of practices around understanding the needs, requirements, and limitations of end-users [7, 10]. It can improve strategic decisions and increase the effectiveness of individual projects and services [10]. The United States Food and Drug Administration (FDA) recently required human factors design and evaluation practices for a wide range of medical technologies [18].

Our research focuses on health prevention by reinforcing healthy habits (e.g., regular physical activity) using an intelligent eCoach system to generate meaningful and personalized lifestyle recommendations automatically. We plan to collect data (personal and activity data) from a healthy group of adult participants, both male and female, over a defined period, followed by the analysis of the time-series data with regards to the impact of lifestyle recommendations on the reinforcement of positive habits of the participants. Also, we plan to collect personal preferences with a self-reporting form and activity data through a wearable activity sensor with minimal burden to the participants. A key goal is to develop a roadmap for self-management with eCoaching that accelerates development, generates best practices, and raises public awareness.

We began with a broader perspective of the ProHealth eCoach system that explored potential designs and development for self-management of behavior (physical activity, nutrition, and habits) focusing on obesity as a study case with the following research questions (RQs):

- *RQ-1: What are the opportunities of an “eCoach application” in eHealth?*
- *RQ-2: What type of goal setting will be needed for self-management of behavior for a healthy lifestyle?*
- *RQ-3: Which feedback from an eCoach to the users would have an effective impact on the have an effective impact on the motivation for self-management of their behavior?*
- *RQ-4: How should the feedback be presented, and what information should be visualized and how?*

Later, we primarily focused on self-management of physical activity for activity coaching. To achieve this, we set the following RQ:

- *RQ-5: How to visualize continuous and discrete data, personalized recommendations, and activity goals in an eCoaching application for physical activity?*

We approached the above RQs by conducting two design workshops during 1 year. These workshops actively involve everyday participants and subject-matter experts in information and communication technologies, health informatics, computer science, nursing, information systems, and HCI. We designed these sessions to explore the challenges and opportunities of eCoaching in self-monitoring, self-management, recommendation generation, and feedback visualization to motivate users to improve their healthy lifestyles. In this paper, we present the methods and results of the workshops, critical insight, and the working research prototype, demonstrating the results of this research as a starting point for digital health monitoring, self-management, and HCI innovation.

### Related work

This section presents existing background knowledge applicable to current research. Different research groups have been conducted different studies related to UCD strategies for technologies that support behavior change for daily life. We considered systematic literature search with the following search string pattern: *((design strategies OR user-centered design) AND (behavior change OR lifestyle) AND (persuasive technology or persuasive strategy) AND (smartphone application OR mobile application OR web-based application) AND (goal-setting or self-management) AND (visualization OR recommendation OR feedback OR notifications))* on the following electronic databases – Scopus, EBSCOhost, ACM, Science Direct, AMIA, JMIR, IEEE, Google Scholar, and Springer. A subset of these articles is cross-referenced between portals, especially Google Scholar and PubMed. Related search keywords were identified using terms of MeSH (Medical Subject Headings), synonyms, relevant articles, and self-determined search terms. We used EndNote (V. X9), DOAJ, Sherpa/Romeo, and Microsoft Excel (MS Office 365 V. 16.x) to efficiently search, collect, and select related articles. We included articles which are peer-reviewed, full-length, and written in English. UCD design approach and its application domain in eHealth is broad. Therefore, the search results have been selective and are further refined to focus on UCD methods, behavioral intervention, lifestyle, and personalized recommendations (see Table 1). From the literature search, the UCD approaches can be classified as follows: iterative, non-iterative (sequential), and other approaches.

**Table 1:** A qualitative comparison between our study and the related studies

Study	UCD Approach and/or Method	Behavioral intervention and purpose	Personalization approach
Our study	Iterative approach	Activity coaching to reduce sedentary behavior	Preference-settings, self-monitoring, interval prediction, and recommendation visualzation
[19]	Iterative approach	To deliver rehabilitation strategies in chronic conditions	Self-management report generation
[20]	Iterative approach	Rural eHealth nutrition education for low-income families	–
[21]	Iterative approach and collaborative engagement	For elderly decision-making towards care location	–
[22]	Iterative approach	Prototyping for a clinical ecosystem	–
[23]	Iterative approach	To support obese and overweight adolescents with a future focus on healthy lifestyle and economic advantage	Feedback presentation
[24]	Iterative approach	Nutritional intervention for healthy lifestyle	Recommendation generation, reminder design
[7, 18]	Non-Iterative approach and Shah’s methodological framework	Physical activity for primary care for patients with chronic obstructive pulmonary disease or type-2 diabetes	Goal-setting and general feedback generation
[25]	Non-iterative approach	To enhance the physical activity level	Goal management, rewards, and self-monitoring
[26]	Non-iterative approach	Proposed and validated the design strategies for persuasive technologies	–
[27]	Non-iterative approach	To design and develop a technology-mediated therapy tool for adults with mental illness	–
[28]	Structured method	For independent and safe elderly living	–
[29]	Participatory design approach	For the self-management of food, exercise, mood, and social values	Graphical representation (e.g., picture and text)
[30, 31]	Evidence-based approach	Remote patient monitoring and early detection of health risks	–
[32]	Behavioral engagement	Behavioral improvement by reducing alcohol consumptions	Daily notification generation and feedback visualizations
[33, 34]	–	Highlighted the importance of self-management for developing the gradual human behavior change intervention strategy	–

### Iterative approach

Richardson et al. [19] adopted an iterative UCD approach to developing a web-based app for delivering rehabilitation strategies (e.g., self-management support and services) with enhanced accessibility, availability, and affordability for the people/participants with chronic health conditions. They developed a prototype with close consultation with rehabilitation experts and performed usability tests, heuristic evaluations, and a target group analysis. Atkinson et al. [20] adopted an iterative UCD approach to developing a rural eHealth nutrition education website for low-income families so that low-income women can use the prototype effectively. The UCD approach focused on – identification of user’s content needs, identification of access concerns, content confirmation, and determination of the functionality, usability, and acceptability study to make the look and feel of the website better. Garvelink et al. [21] adopted a three-cycle (iterative) UCD approach for elderly decision-making towards care location. The cycle consisted of the following steps – cycle 1: ideation and requirement gathering on home-care service delivery and the development of the prototype based on the input from the end-users, cycle 2: usability testing with end-users and re-design, and cycle 3: final refinement with a linguist, graphic designer, and end-users. The result shows a successful design and development of a decision guide system for the elderly population with a fully collaborative approach. Pais et al. [22] performed an iterative UCD study to develop a proof-of-concept prototype for a clinical ecosystem that can integrate, and store health and wellness data generated by commercially available mobile apps in Gestational Diabetes Mellitus (GDM) care. The UCD approach helped them gather end-user requirements and refine them further in successive iterations to meet the expectations of end-users. LeRouge et al. [23] performed a qualitative user-centered design study to design a technology-mediated nutritional program to support obese and overweight adolescents with a future focus on money savings (or economic advantage), healthier dietary planning, good societal impact, and enhanced self-efficacy. They divided the UCD approach into two iterations – early-stage prototype usability analysis and semi-structured interviews with health professionals and end-users. The result produced good reflections on existing theories about personalized behavior change and design requirements for feedback presentation (e.g., vivid colors, semirealistic images, cooking sounds, multimedia, and gaming). Mummah et al. [24] conducted an iterative UCD approach to design “Vegethon” to perform a theory-based smartphone app-based nutritional intervention with an IDEAS framework to enhance vegetable consumption. The key findings were – focus on self-monitoring, the inclusion of challenges, simplified features (e.g., weekly reporting), goal setting, recommendation framing, effective recommendation generation, reminder design, and evaluation.

### Non-iterative approach

Van der Weegen et al. [7] and Verwey et al. [18] conducted a user-centered design study to develop a smartphone-based monitoring and feedback generation tool to simulate patients’ physical activity with lifestyle diseases. They followed Shah’s methodological framework in three iterations for medical tool development. The study demonstrated how the user-centered design approach helped integrate concepts, such as literature findings, tool architecture, goal setting, feedback generation, feedback visualization, data sharing, and consequences into a smartphone app and mature it further with iterations. Munson et al. [25] performed a user-based study with their developed mobile phone app, “GoalPost”, and “GoalLine” to better understand the impact of goal setting, rewards, self-monitoring, and sharing of goals, goal progress on enhancing the physical activity level. The study found that generation of secondary and primary objectives and non-judgmental reminders were effective among participants; however, rewards must be designed more effectively for more participant engagement. Consolvo et al. [26] proposed and validated the design strategies (e.g., abstract, and reflective, unobtrusive, public, aesthetic, positive, controllable, trending, and comprehensive) for persuasive technologies using a user-based study to motivate and help people/participants in improving their daily negative behavior with technology design. Lederman et al. [27] adopted a three-month user study to design and develop a technology-mediated therapy tool for adults with mental illness, including psychoeducation, therapist moderators, social networking, goal-based analysis, and HCI approaches. They found that an effective engagement of end-users with automated system behavior and the development of therapeutic alliances are essential for mental health therapy and self-determination theory.

### Other approaches

Harte et al. [28] successfully derived a structured methodology (three-phase method) with the UCD approach to design and develop a health system, namely “Wireless Insole for Independent and Safe Elderly Living (WIISEL)” for elderly care with fall-risk prediction. In phase 1, they focused on creating use case documents using storyboarding, paper prototypes, mock-ups, and user interviews. Phase 2 focused on expert usability inspections (e.g., heuristic evaluations, prototype review, and feedback generation). In Phase 3, they did classical user testing with user experience to improve the final *WIISEL* prototype. Kim et al. [29] performed a user-centered participatory design approach to design and develop an iOS application for the self-management of food, exercise, mood, and social values in the form of pictures and texts for obese or overweight adults. The study showed promising results on enhancing self-awareness towards a healthy lifestyle and behavioral change, effective engagement, and self-reporting to manage the factors that impact obesity or overweight. McCurdie et al. [30] and Bruce et al. [31] adopted a UCD evidence-based approach to develop a mHealth consumer app to enhance healthcare delivery and clinical outcomes with remote patient monitoring and early detection of health risks to avoid severe damages. Their research reveals the importance of a user-centered/patient-centered approach in achieving user engagement (or user evaluation) to enhance the effectiveness of behavioral interventions. Bell et al. [32] explored how user engagement can improve the research and development of a behavior change app iteratively. They performed a behavioral engagement longitudinal observational study on a group of participants using their “Drink Less” behavior change app for alcohol reduction and explored parameters, such as frequency, amount, depth, and duration of the study with a simple data visualization tool to understand user psychology to improve the app further. Branford et al. [33] also emphasized the importance of privacy, trust, and experience, as well as opportunities to provide healthcare and empower people to manage their health and well-being in a way more suited to their lives and values ​​to design and develop HCI-based digital health technologies effectively. Araújo-Soares et al. [34] highlighted the importance of self-management for developing the gradual human behavior change intervention strategy in chronic diseases. Interventions that address risk factors and support behavioral changes to effectively self-manage chronic disorders can significantly impact health and well-being and reduce the cost of providing medical care for the elderly with chronic diseases [34]. Self-management is a complex task, including adherence to therapy, changing multiple health behaviors, and regular contact with healthcare providers [34]. Interventions often include additional components to establish and maintain harmony and participation through interpersonal communication methods or functions, such as gamification in digital health interventions [34]. The development of healthy behavior change interventions determines the best combination of these characteristics and the transparent reporting of these decisions [34].

Therefore, an iterative UCD approach has been essential to address the users’ health requirements and the technological needs throughout development and to design and develop personalized care applications in the healthcare domain to increase its acceptability, credibility, and effectiveness. Human behavior is crucial for customized care, particularly in chronic illnesses [35]. It requires the effective engagement of end-users, proper recommendation generation, and its presentation. The *state-of-the-art* of this research is input to the design and development of an initial working prototype of an eCoach mobile app that can recommend, and motivate participants with personalized recommendation messages and its meaningful presentation. Our mobile app design follows the standard “Google Material Design” guidelines. The design provides with usable themes, material guidelines, system icons, and color palettes to craft an intuitive eCoach app. In this study, our focus of eCoaching has been physical activity coaching; however, its scope is not limited to that.

Many e-coaching apps offer personalisation features in the market. However, they are missing to have multiple generic eCoaching components. The motivation of this study is to design and develop an initial workable research prototype using an iterative design process for personalized recommendation generation and meaningful representation. Moreover, we provide a further innovative direction to research how AI technology could be utilised for effective recommendation generation. A qualitative comparison between our study and the related studies has been made in Table 1 based on the following three parameters: UCD approach and/or method, behavioral intervention and purpose, and personalization approach.

## Methods

We have followed the Standards for Reporting Implementation (StaRI) for this comprehensive UCD study (see Additional file 1). All methods have been carried out in accordance with relevant guidelines and regulations in the “Ethics approval and consent to participate” section under Declarations.

### User-centered Design approach

A standard UCD is an iterative process with four phases: understand context of user, specify user requirements, design solutions and thereby, evaluate design against requirements. The aim of the UCD process is to capture and address the whole user experience with an explicit understanding of the users, tasks and environments [11–13]. We have used such an iterative design process to shape the eCoach prototype design with the end-users. Therefore, the user’s context and expectations from a computerized system must be well understood to increase the accomplishment rate in functional testing, acceptance testing, usability testing, and credibility testing. To facilitate UCD from the beginning, we involved subject-matter experts with a background in HCI, health informatics, and computer science in gathering knowledge on the design approach’s needs, demands, and restrictions. We conducted a preparation meeting before design workshop 1 to exchange expert’s knowledge, thoughts, and ideas from their experience. These experts delivered feedback on the importance of methodology selection, use case design, identification of the background of participants to be recruited as end-users in order to avoid design biasness, fair distribution of participants among different groups, time planning for conducting a digital workshop, potential distractions in digital workshops, challenges with effective engagement, questionnaire design, comprehensibility of the questionnaire and required consent for participant recruitment. We identified the importance of a moderator who can moderate the digital group discussions and involve all participants in each group.

We planned the entire process to be user-friendly and interactive. The future objective of using this eCoaching app is to perform a usability study followed by a longitudinal study on a controlled group of participants to verify the practical effectiveness of using this app toward a healthy lifestyle with self-management, self-motivation, and self-correlation. Therefore, in our study, end-users are a potential subset of actual eCoach participants for our future studies. We aimed at involving end-users in the early eCoach design phase to understand their thinking and expectations to avoid further design conflicts. The working domain and the area of expertise of each end-user can be diverse. Therefore, we decided to recruit participants from the following occupations – student, researcher, health professional, educationalist, and IT professional to bring diversity to the workshops. Our study targeted participants with standard body mass (BMI) ranges [18.5–25 kg/m2] as well as obese and overweight [25–35 kg/m2]. The initial selection criteria are described below:

### Exclusion criteria

- Participants who do not have wif-fi or wireless broadband (BB) at home.
- Participants with postcode outside Southern Norway.
- Partciapnts with severe medical history (illness, hospitalization) conditions) of last one year and sever chronic health issues that may interfere with appropriate data collection.
- Participants with food allergies.
- Underweight (BMI < 18.5).

### Inclusion criteria

- Participants registered to general practitioner (GP).
- Age group 18–64 with targeted BMI range.
- Participants having wif-fi or wireless BB at home.
- Participants in South-Norway.
- Participants motivated for self-monitoring and data collection.
- Participants without a prescribed major chronic condition or current disease episode.
- Can speak, write, and read English in an understandable way.

**Fig. 1:**
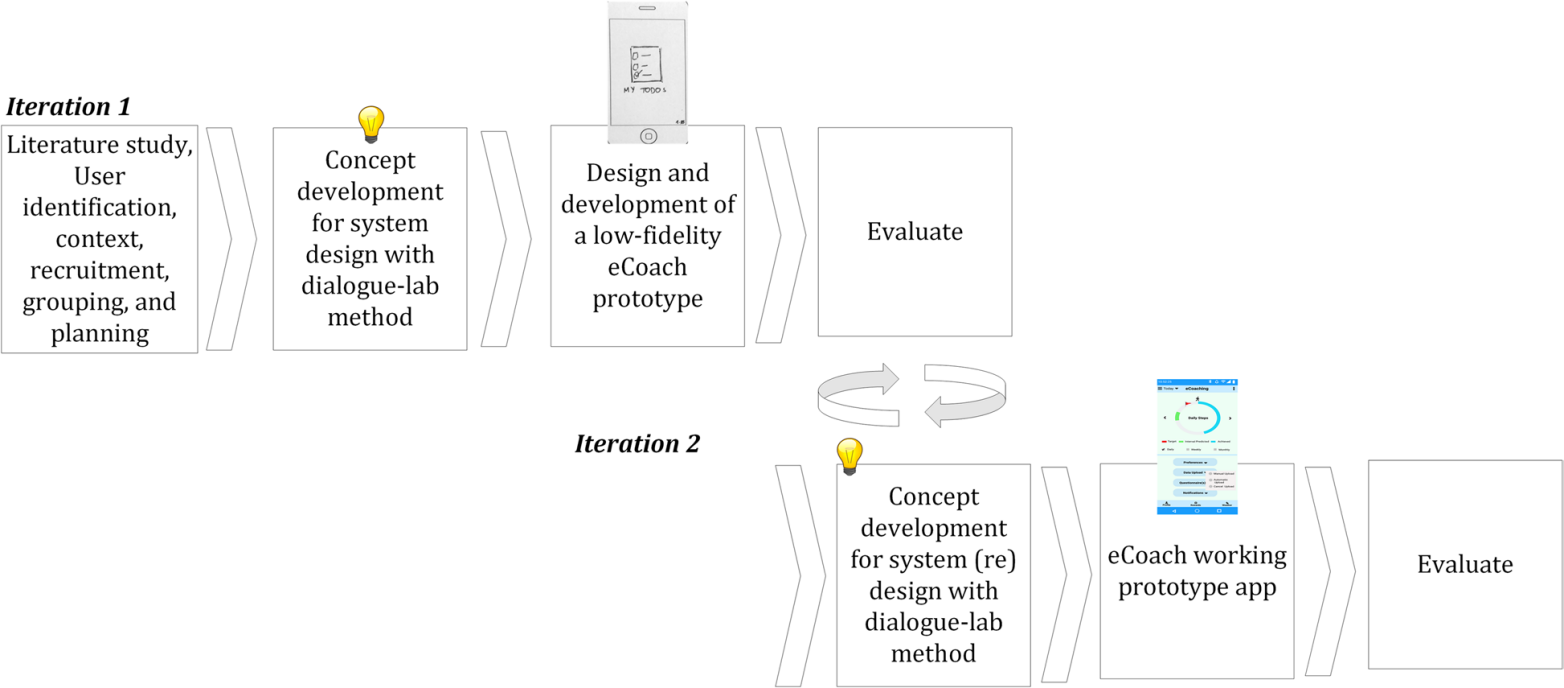
Adopted process for the iterative-user-centered eCoach prototype design and development

We agreed to adopt an “iterative” UCD approach [7, 11, 12, 36]. The adopted strategy follows iterative stages (identify end-users and their context, concept development, design, and prototype development to establish the recognized concept in the mobile app) as depicted in Fig. 1. We decided to start the workshop with an introductory presentation of the objective and the motivation for conducting such a project to give the participants a high-level overview. The entire workshops have been conducted digitally using zoom due to the COVID-19 pandemic. All the video and audio recordings were maintained following the data security and privacy guidelines set by the Norwegian Centre for Research Data (NSD) [37].

At the end of workshop 1 (iteration 1), the research team collected all user needs and preferences. An engineering team helped translate the needs into initial technical solution requirements. The initial solution was developed based on continuous interaction and feedback generation between the engineering team and the research team. Afterward, the initial solution was utilized in workshop 2 (iteration 2) to gather feedback on the gap between the technical solution and the user requirements (particularly regarding the visualization of physical activity recommendations). The result of the second iteration helped us to mature the design and the technical solution.

### Workshop 1 –Design and end-users

#### User identification, context, recruitment, grouping, and planning

We ran workshop 1 to apply eCoaching for an obesity and overweight risk target group to promote a healthy lifestyle through behavior monitoring and personalized recommendation generation. The aim of eCoaching was healthy and obese (or overweight) participants in good health, men and women between 18 and 64. A primary objective of this workshop was to focus on the study’s goal (i.e., develop a roadmap for self-management with eCoaching that accelerates development, generates best practices, and raises public awareness). During the workshop, we explored the opportunities and challenges of eCoaching with participants and experts on the first four RQs. The workshop structure was based on the dialogue-labs methods from Lucero et al. [17], which facilitate the generation of participants’ ideas by stimulating their creative thinking through a sequence of design activities.

Eight end-user volunteers (7 M; 1F) aged 18–64 (student (1), research scholar (1), health professional (1), educationalist (4), IT professional (1)) and four experts (4 M; 0F) aged 18–64 participated in the workshop along with three facilitators from the research team. The workshop was executed online (via videoconferencing tool “Zoom”) at the University of Agder, Norway. We divided the end-users randomly among four groups headed by one expert (E). We created a Zoom breakout room for each group to discuss and exchange their thoughts with experts. We asked each group to present their ideas using a shared online whiteboard.

We then gave a 20-minute slide-deck presentation using images, the concept of eCoaching, and a brief introduction of the project with objective and motivation. We split volunteers randomly into four groups (2 × 4 people, 1 × 4 experts) and these groups took part in two activities. In each group, experts kept advocating the reasoning of the participants by asking the incitement questions. We created two Flyers as props for conducting workshop 1. Flyer 1 includes workshop design and development details, which we shared with potential end-user groups before the workshop. Flyer 2 includes detailed activities of the workshop for experts.

##### Task 1 (30 minutes)

We distributed RQ1 and RQ2 to all four groups and brainstorm with groups in the first 20 mins. Then we asked groups to present their ideas with other groups in the last 10 mins.

##### Task 2 (30 minutes)

Like Task 1, we distributed RQ3 and RQ4 to all four groups and brainstorm for in first 20 mins. Then we asked groups to present their ideas with other groups in the last 10 mins.

### Concept development for system Design

In this workshop, we have integrated the identified behavior change strategies and technologies in the eCoaching prototype design to stimulate a healthy lifestyle (physical activity, proper diet, and healthy habit) corresponding to users’ context and needs (overweight and obesity risk management).

In the preparation phase, we discussed user and context description, our literature findings, and the study’s objective with experts to plan and create tasks for the workshop 1. We provided the users with a high-level conceptual idea about the health eCoach system and its objective in the obesity study case without disclosing too many details about the research and engineering thoughts. It helped end-users to brainstorm their ideas regarding the targeted research questions. Workshop 1 helped us develop general use cases to describe the interaction between the eCoach prototype system to be developed and the end-users in a stepwise approach. After the experts’ focus group discussions and result presentations, we created the first draft of a user requirement document to modify it further in the next iteration to reduce the design, development, and user expectation gaps. The researchers and the engineering team worked on the first draft to do a feasibility (technical and financial) check to ensure no significant issues were missed. We created an eCoach prototype app as an initial solution based on the first draft of user requirements to improve it further based on the feedback from end-users in the next iteration.

### Evaluation of the ProHealth eCoach as a low-Fidelity prototype

The low-fidelity eCoach prototype for activity monitoring as an outcome of workshop 1 was presented to the end-user groups in Workshop 2 to receive valuable feedbacks. The feedback consisted of three choices – passed (5), failed (0), and further scope of improvement (3). We demonstrated the followings to evaluate the prototype –

- Selection of activity sensor, its wearing, and its connection establishment process with the app for the collection of data.
- Information to be collected for authentication in the login page.
- Set of preference data to be collected in the form of questionnaire.
- Prepared questionnaire set for the feedback or suvey, and reporting of technical problems during study in progress.
- Layout, content, icon, and color selection for the following pages: homepage/login page, data upload, preferences, questionnaire, notification and reward generation.
- Visualization layout for daily, weekly, and monthly activity patterns.

We used Figma app view, basic web page view, and PowerPoint for the demonstration. We received feedback for email id-based login instead of long a unique user identifier or UUID-based login, different modes of data upload from the activity sensor, refining questionnaires sets and their design, uniform layout design, and selecting a standard color with appropriate icons for each eCoach views and concepts, and an integrated circular layout for visualizing activity patterns over time. Overall, we received an average feedback rating of 3.0 out of 5.0. All the feedbacks or comments were addressed in the initial eCoach working prototype app.

### Workshop 2 – (re) Design and end-users

#### User identification, recruitment, grouping, and planning

We created two Flyers as props for conducting workshop 2. Flyer 2 includes workshop design and development details, which we shared with potential end-user groups before the workshop. Flyer 2 includes detailed activities of the workshop for experts.

We ran workshop 2 to bring together different types of users, such as non-technical, technical, subject-matter experts, familiar participants, and people from cross-domains, to join a creative process for making the eCoach prototype attractive, persuasive, easy to use, and suitable for daily use. A key goal of this workshop was to generate ideas to improve the quality of the personalized feedback, the visualization of self-monitoring data and recommendations, and goal setting. During the workshop, we explored the opportunities and challenges of eCoaching with participants and experts regarding the RQ5. The structure of this workshop was also based on the dialogue-labs methods from Lucero et al. [17], which facilitate participants’ generation of ideas by stimulating their creative thinking through a sequence of design activities.

We host the workshop online (via Zoom) at the University of Agder, Norway. Nine end-user volunteers (8 M; 1F) aged 18–64 (student (2), research scholar (2), health professional (1), educationalist (2), IT professional (2)) and four experts (3 M; 0F) aged 18–64 participated in the workshop along with three facilitators from the research team. We divided the end-users randomly among four groups headed by one expert (E). We created a Zoom breakout room for each group to discuss and exchange their thoughts with experts. We asked each group to present their ideas using any virtual whiteboard.

We then gave a 5-minute slide-deck presentation using images, a brief introduction of the project with objective and motivation. We split volunteers randomly into three groups (3 × 3 people, 1 × 3 experts), and these groups took part in the respective activity. In each group, experts kept advocating the reasoning of the participants by asking the incitement questions.

##### Task 1 (30 minutes)

We distributed three sub-research questions under RQ5 to all three groups and brainstorm with groups in the first 15 mins. Then we asked groups to present their ideas with other groups in the last 15 mins. We then gave a 5-minute slide-deck presentation using images, a brief demonstration of initial version of eCoach app.

##### Task 2 (30 minutes)

Like Task 1, we distribute the topic on “feedback generation and presentation” to all three groups and brainstorm for in first 10 mins. Then we asked groups to present their ideas with other groups in the last 10 mins. In the end, we did a plenary discussion for 10 mins.

#### Concept development for system (re)Design

Workshop 2 (iteration 2) helped us to collect users input for the improvement of the quality of goal settings, motivational status visualization from self-monitoring, personalized feedback generation based on artificial intelligence (AI) technology, and recommendation visualization. The focus of this workshop - was on the personal preferences. In this context, we reformulated RQ-5 and prepared the following sub-questionnaires for preference(s):

##### Goal setting

- What goals do you want to set for activity coaching (e.g., nature of goals)?
- How to inform about goals (e.g, direct vs motivational)?
- How to set the goals (e.g., generic vs personalized)?

##### Response type and coaching

- What goals do you want to set for activity coaching (e.g., nature of goals)?

##### Interaction type

- How do you want to interact with the eCoach?
- Mode (style, graph)
- Frequency (e.g, hourly, quarterly, once, twice)
- Medium (e.g., audio, voice, text)

We divided the topics of discussion, such as “Goal Setting”, “Response and Coaching”, and “Interaction” between three groups led by experts. End-users were motivated to draw an intended design for the data presentation and recommendation visualization to use online worksheets. This workshop helped us to narrow down the scope of eCoaching from a broad area of behavioral coaching to only physical activity coaching to reduce sedentary behavior. Collected feedback from the end-users and experts provided ideas to (re) design the initial holistic eCoach prototype towards the development of an activity coaching mobile app based on selective considerations.

#### Evaluation of functional Design of the Initial Working eCoach prototype app

We invited the same end-users and observers to evaluate the functional design and working of the initial eCoach prototype app with heuristic approach and give feedback. We handed over the prototype to each group to do a hands-on functional testing under our lab settings and the outcomes are noted in Table 2 as a form of feedback. The feedback consisted of three choices – passed (5), failed (0), and further scope of improvement (3). We received a rating of ≈ 4.1 out of 5.0 with further improvement scope in layout design to give the app a sophisticated view.

**Table 2:** Feedback results of functional testing on the initial working ProHealth eCoach prototype

Feedback	Choice
Simple email-based login	Passed
Simple connection with activity sensor	Passed
No problem with using the activity sensor	Passed
Successful collection of data with sensor	Passed
Successful collection of data with questionnaire	Passed
Page layout design	Further scope of improvement
Proper color in page design and icons	Further scope of improvement
Easy to navigate	Passed
Data visualization is easy to comprehend	Passed
Correct word and letter selection	Further scope of improvement
Proper alignments	Further scope of improvement
Notification delivery	Further scope of improvement
Reward planning and visualization	Further scope of improvement
Version Compatibility	Further scope of improvement
Page loading time	Passed

### Data capture and analysis

During the design workshops, researchers collected data with text notes (using Notepad++, Google Docs, and digital Sticky Notes), video, and images. We prepared two separate folders (for two iterations) in Microsoft Teams to store the materials safely with access control rules. At the end of the first workshop, materials from respective folders were assembled and analyzed to understand themes and categories. Also, we discussed and refined our understanding with the research team. We synthesized the most general scenarios and interaction styles. We used workshop 1 as an input to the next workshop. The data from workshop 2 helped to refine the design and implementation of the working research prototype of eCoach system.

## Results

This section describes in detail the results from (a.) workshop 1 (iteration 1), (b.) workshop 2 (iteration 2), and subsequently, (c.) the overall design considerations based on the workshops to develop working research prototype of eCoach for personalized activity recommendation generation.

### Workshop 1

#### Iteration 1: scenario Design

From workshop 1 we identified end-users and their context and followed by, developed a concept (user requirements) based on the focus group discussion to answer the identified research questions as described in Additional file 2-5.

Despite the initial briefing by our team about the motivation of a health eCoach app, all groups suggested that eCoaching must be user-friendly, accessible, effective, evidence-based, predictive, transparent, and accurate. In goal settings, end-users told that goals could be open, flexible, adjustable, specific, measurable, attainable, relevant, sharable, and real-time. One group suggested considering cultural aspects, social traits, and individual preferences regarding coaching or motivating. The other two groups suggested the inclusion of key performance indicators (e.g., an overall health index computed by combining several health parameters to forecast health status and update the timely progress indicator) in automatic lifestyle coaching.

All groups suggested that long-term goals must consist of multiple short-term goals, daily goals must be different from long-term goals, and personal preference based. An individual will be motivated if rewards, performance comparison, constructive motivational feedback, and personal preferences are incorporated in eCoaching. One group suggested including gamification, mood assessment, and iconography to convey feedback without requiring much cognitive involvement of the user. The other two groups ideated to consider a progress evaluation graph or report, fitness status evaluation, goal comparison, timing feedback, reminder design, and high-level contextual information in feedback generation to motivate participants in self-management.

#### Discussion: concept Design for Personalized Recommendation Generation

The discussion opened a broad scope for the eCoach system to promote a healthy lifestyle. Usability, credibility, and effectiveness were identified as essential factors to determine the performance of an eCoach system. According to the discussion, the needed data collection for activity, nutrition, and habit is necessary without burdening the participants. Personalized goal setting, health risk prediction, goal evaluation, and evidence-based contextual real-time tailored recommendation generation are essential features for health eCoaching. Goals must be intelligent, customizable, personalized, and context-driven in goal settings. Iterative recommendation generation based on health status adjustment, reminder design, adjustable preferences, progress evaluation, rewarding, realistic feedback generation, and an appropriate information visualization may motivate participants to self-monitor and manage their goals. Recommendation generation can be combined with personalized mood assessment feedback to determine the satisfaction level of participants. The eCoach app must exhibit beyond state-of-the-art innovation to be better than existing apps to manage individual behavioral change. This workshop helped to refine the questionnaire set in the eCoach prototype design and development for meaningful, personalized recommendation generation.

#### End-user’s remark on personalized recommendation generation

*“My FITBIT scares me a bit, because it constantly tells me that I sleep too little. It is perceived as annoying bullying and I cannot set up that I do not want all this feedback. My experience is that I like to see that I have been active from week to week, and I probably think that I am more conscious, and that it motivates me to make the right choices”.*

We created a basic initial eCoach prototype for personalized activity coaching given by the participant’s discussion and design to capture the high-level plan for goal management and tailored recommendation generation in activity coaching and interactions predicted across groups. Researchers involved in workshop 1 created an eCoach prototype over the next month using data and objects of the workshop. The prototype was further modified based on the outcome of Workshop 2.

### Workshop 2

#### Iteration 2: scenario co-(re)Design

We started the workshop with a group discussion focusing on preference(s) and motivation. The selected topics were – goal settings, response and coaching, and interaction type.

According to group-1, goals can be generic as well as personalized. In our eCoaching, personalized goal management will be more meaningful than the existing market apps. They addressed that goal setting is an essential aspect in eCoaching. Goals can be set up by a doctor, a nurse, or a person. Thus, a contextual consideration is necessary for the eCoach design and development. As suggested, goals must be broken down into more detailed, specific goals linked to the more significant life priorities social and competitive perspectives. Group 2 indicated that motivations could be - user-based, situational-based, and environmental-based. An evidence-based personalized recommendation generation strategy will be very relevant for our eCoaching. According to group 2, selection of appropriate target group, presentation of data, selection of the device, type recommendations, and innovative motivational feedback presentation are essential in our automatic activity coaching with the choice of feedback generation frequency. Group 3 highlighted that interaction type is highly related to “user type” and their “emotional state or perspective”. The interaction design in our eCoaching must be two-way, adaptable, ubiquitous, easy to comprehend and visualize, accessible, customized, and personalized.

#### Discussion: preference settings for personalized recommendation planning

From workshop 2 we gathered end-user feedback on the personal preference settings (goal settings, response type for coaching, and interaction type) for personalized recommendation generation and visualization in a health eCoach app based on the focus group discussion to address the RQ5 and its sub-questions. In this workshop, we narrowed the scope of holistic behavioral coaching for managing body weight to only activity coaching to reduce sedentary time.

In goal settings, the goals can be personalized or generic. The generic goals in activity coaching can be general activity guidelines set by the WHO [6, 7]. In contrast, athletes or obese or overweight people who want to stay active or reduce their weight to a normal range can set the goal, differing from WHO’s generic guidelines. The personalized activity goals can be multiple types (e.g., weight reduction, staying active, body fat level, proper sleeping) and need prioritization. Besides the selection of goal types, goal setting is also essential. A question may arise who will set the goals: a doctor or a trainer or the person itself! However, it depends on the context. Goal scoping in context is also an essential factor in effective coaching. Therefore, it should be broken down in a more readable, detailed, and specific way to link to the purpose. Besides, in successful goal management, social or community perspective (e.g., doing activities together) and/or competitive views (e.g., ranking, rewards) should be addressed. Overall, goals shall be “SMART”: specific, measurable, attainable, relevant, and time-bound.

Motivation is the desire to take action to achieve a goal. It is a critical factor in setting and achieving goals. Motivation is one of the driving forces behind human behavior. It includes the desire to continue working towards meaning, a purpose, and a life worth living. In eCoaching, motivation is an essential factor in daily life activities. Motivation differs from person to person based on the context (e.g., feedback generation to motivate a blind participant differs from a non-blind or color-blind participant). Participants can be encouraged with personalized, evidence-based, and contextual recommendation generation and its purposeful presentation (e.g., graphs, selection of colors, contrasts, visual aspects of movements, menus, adjustable with device type). Charts can produce a visible reflection of time-bound activities; however, app developers should consider the device’s battery usage.

Interaction is an action that occurs due to the mutual influence of two or more objects. The concept of two-way effects is essential in interaction, not one-way causal effects. The factors associated with a good interaction design are – two-way interaction (e.g., having a diologue), ubiquitous interaction (e.g, interaction at home, outside, office or in running or walking), opportunistic (e.g., triggered automatically), adapted to the situation (e.g., former activity the user was doing at the same place, time frame, adaptive in some way based on user’s instructions (e.g., visual, audible)) or, interaction preferences (e.g., user needs to see anything, only hear something, feel something, emotional needs, understanding (e.g., complexity), and motivated), visualization of graphs (e.g., what will you use the graphs/voice for?), frequency of interaction (e.g., hourly, twice/thrice per day, per day, weekly, bi-weekly, monthly), accessibility (e.g., voice, chart or graph, text to speech, text), situation awareness (e.g., situation awareness, multimodal interaction), usable and accessible following the international standards, culturally adapted following the cultural conventions, error reduction by design (e.g., redundancy), and personality (e.g., type of user and their action). Notification generation and presentation are a part of interaction and can be persistent or not. In notification design, a balance should be maintained between relevance, persistency, and disruption.

#### End-user’s remark on motivation

“I wish to expend 7*X (X>0) calories per week. I can spend more than X calories on a day when I am highly motivated. Then, it would be nice if the system saves the extra calorie expenditure in a virtual energy bank that I can expend on a lower motivated day or treat myself to my favorite food (e.g., a chicken burger).”

“*The app should generate contextual recommendations to motivate. Example – I am highly interested in soccer, and the app knows it. While I am walking or running, the app can track if any soccer event is progressing nearby and can recommend me with a message like if you walk or run X kilometers, then you have a chance to enjoy an exciting soccer game.*”

#### End-user’s remark on feedback generation

*“Daily feedback would be better instead of every minute or hour.”*

*“Personalized activity recommendation should be presented in the form specialized graph or chart based on activity, goal setting and goal achievement to motivate participants.”*

*“Feedback could be internal or external. Internal feedbacks should be generated through the device or eCoach app. External feedbacks can be generated from external sources.”*

#### End-user’s remark on interaction

*“Graphs: for someone without academic background or low graphical literacy, how do they understand? May other forms of interaction from the eCoach app.”*

*“Think of presentation of graphs: understand the level of literacy when having visual text. It is widespread. It is important to think very clearly about various questions. What is the goal of the graph? What type of information is needed? How can they adapt of different levels of literacy (e.g., visual numeric literacy)? Is it possible to have different shapes and forms and screen sizes?”*

*“One notification every one hour may be too disruptive.”*

We presented the initial activity eCoach prototype in workshop 2. We received participants feedback to improve the quality of goal settings, motivational status of visualization, personalized feedback, and recommendations. The overall design and modular implementation of the ProHealth eCoach prototype is described in the following sub-section.

### Design and development of the ProHealth coach prototype

**Fig. 2:**
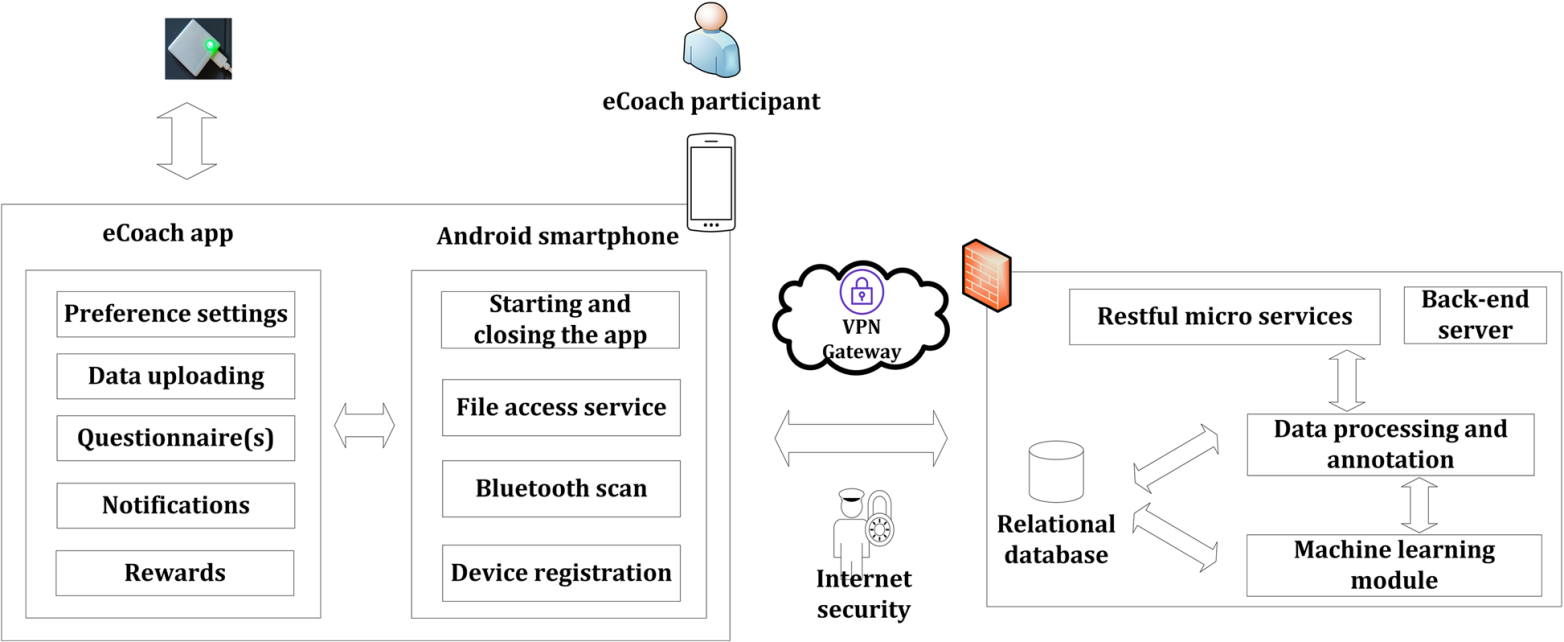
The software development architecture of activity eCoaching app

Here, we describe the high-level design consideration for the ProHealth eCoach prototype. Workshop 1 has given an overview of the necessary data to be collected from the participants relevant to our research’s goal. Workshop 2 helped preference setting, recommendation generation, and its visualization. Our developed ProHealth eCoach app consists of the multiple modules described in Table 3, and the corresponding data considered for prototype design is shown in Table 4. The software architecture of the ProHealth eCoach app development is depicted in Fig. 2. Please refer to the video (see Additional file 6) to see the demonstrators in action.

**Table 3:** Multiple modules of the activity eCoaching app

Module	Purpose
Data Sharing	For user log-in, personalized configuration for activity sensors.
Data Collection	For the collection of sensor data, contextual weather data, and self-reporting questionnaire data.
Preference Settings	For collecting user preferences and persist them. Users can set long term or short-term physical activity goals, or the system can suggest them for a system-defined goal set. Users can edit and change the goals when they want. The level of goals gradually increases with the progress of individual performance.
Monitoring	For AI or rule-based prediction of health state of the participant and compare it with pre-set user goals to generate personalized recommendations. This module also monitors contextual weather data that helps in contextual recommendation generation.
Recommendation Visualization	For visual reflection of activity in progress and displaying future predictions to motivate individuals.
Rewards	For classifying the user’s progress to reach personalized goal at the end of pre-set period into three groups – well done ( ), up-to the mark ( ), must be improved ( ).
Notification or Reminders	For generating personalized reminders adaptively based on the context, preferences, and health state. It can be an audio notification or a push notification with a precise and dynamic content.
Problem Reporting	For addressing technical problems confronted by end-users.

**Table 4:** Data considered to design the activity eCoaching app

Data type	Nature of data	Data
Activity data	Wearable sensory data	Timestamp, steps, low physical activity (LPA), medium physical activity (MPA), vigorous physical activity (VPA), sedentary, weight bearing, standing
Contextual data	External sensory data	Timestamp, city, country, weather code, status, description, temp, real_feel, pressure, humidity, visibility, wind_speed
Goal data	Questionnaire-based preference data	Generic (e.g., system defined) or personalized
Response data	Questionnaire-based preference data	Recommendation data for activity
Interaction data	Questionnaire-based preference data	Mode (e.g., style, graph), frequency (e.g., hourly, quarterly, twice a day, daily, bi-weekly, weekly, monthly), medium (e.g., text)

**Fig. 3:**
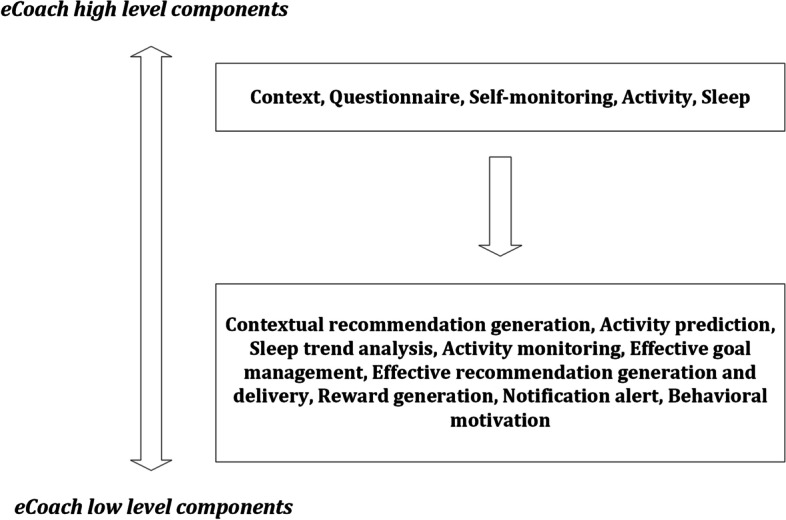
Components of the activity eCoaching framework

On a conceptual level, the activity eCoaching framework consists of – a. high-level components (e.g., activity monitoring, sleep monitoring, monitoring based on self-reports) and b. low-level components (e.g., step prediction, sleep trend analysis, determination of good goal, effective feedback generation for behavioral motivation) as depicted in Fig. 3.

Participants can select single or multiple high-level component blocks for eCoach-based self-monitoring and recommendation generation. In the framework, a semantic ontology can be used to transform distributed, heterogenous health and wellness data (e.g., sensor, self-reported questionnaire) into meaningful information, including health state prediction [3]. We have considered activity monitoring based on time-series data processing with deep learning networks [38]. Here, we presented activity prediction as a set of numbers or intervals and used its visualization for motivational purposes. However, the usability study and the efficacy evaluation of the eCoach app for behavioral motivation is the future scope of research. In our design consideration, the eCoach system has access to contextual weather data, activity sensor data, and questionnaire data. The overall modularized eCoach app design and its implementation is described below, addressing ideas and concerns.

#### Data sharing

**Fig. 4:**
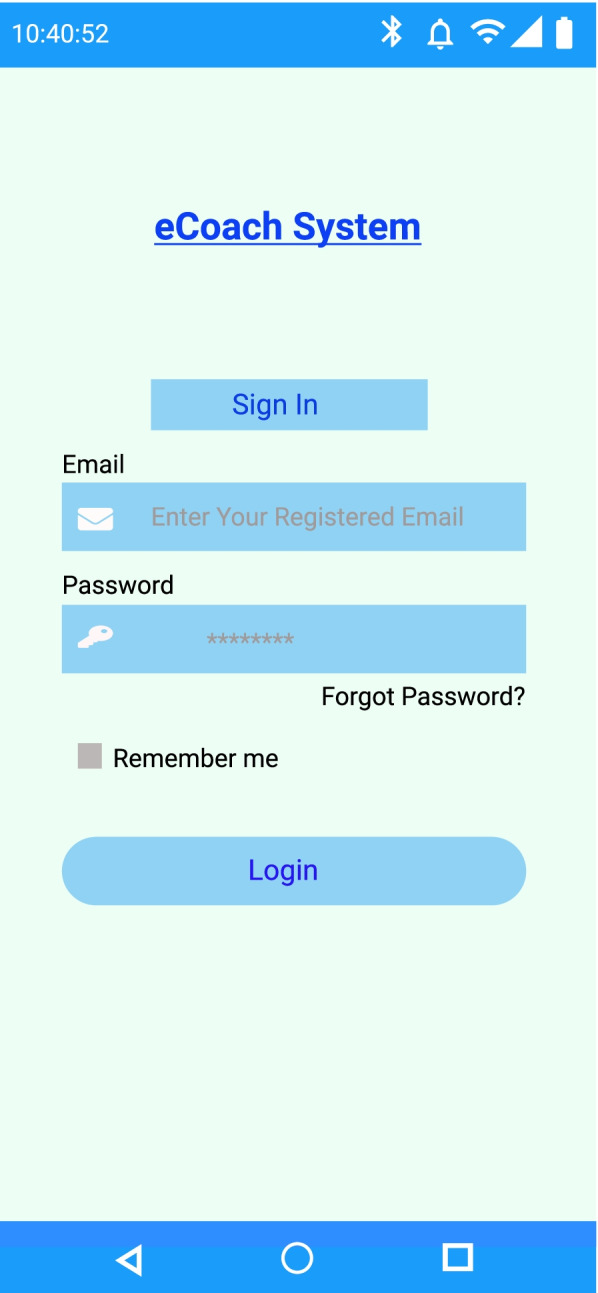
Simple log-in page for the eCoach prototype system

The log-in has been kept as simple and secure as possible. We have planned to collect person-related and activity data without personal identity disclosure. Only authorized users can access the eCoach system. Each participant has been provided with a unique user identifier (UUID), and they will be able to access the system with personal email-id and modifiable password. The system is further protected with the “eduVPN” network. Activity data can only be shared with the researchers to create meaningful information out of raw data. Sharing data through social media or any other means is prohibited by NSD rules. The simple log-in interface of the eCoach app is depicted in Fig. 4.

#### Data collection with eCoach system prototype

The data collection has been divided into four parts –

- Activity data collection with wearable Bluetooth enabled (BLE) low energy activity device,
- Questionnaire for daily weight reporting (to analyze over a period of time whether activity coaching has an impact or not!), feedback (or survey), and the reporting of technical problems (without personal identity disclosure) during study in progress,
- Personal preference settings (goal-settings, response, and interaction), and
- Contextual weather data collection with OpenWeather representational state transfer (REST) application programming interface (API) against API Key validation.

We used the MOX2 medical-grade (CE certified) accelerometer-based low energy activity sensor for continuous monitoring [39, 40]. The device flawlessly measures and transfers high-resolution activity data, such as activity intensity, weight-bearing, sedentary, standing, low physical activity (LPA), medium physical activity (MPA), vigorous physical activity (VPA), and steps for every minute. The collected data is well suited for physical activity classification (LPA, MPA, VPA) and posture detection (sedentary, (such as sitting or lying), standing, and weight-bearing). The recommended wear locations of the device are thigh, hip, arm, or sacrum. We used the publicly downloadable Android MOX2 mobile app to capture individuals’ activity parameters into the smartphone’s download folder. We then used our developed eCoach app to periodically transfer the activity data to the eCoach backend server tagged with the unique user-id, following the android secure file access policy. Participants had the following two options to upload their activity data from their smartphone to the remote eCoach server – automatic (to upload data automatically after every regular interval) or manual (if automatic data upload fails due to technical problems). The personal health, wellness, and questionnaire data are sent from eCoach app to remote eCoach server via a REST API (HTTP POST) to store them in a Postgres database in line with General Data Protection Regulation (GDPR) and Norm for information security and privacy in health (NORMEN) guidelines. No disclosable personal identifier has been collected with the questionnaire, complaint, or feedback (survey) data.

**Fig. 5:**
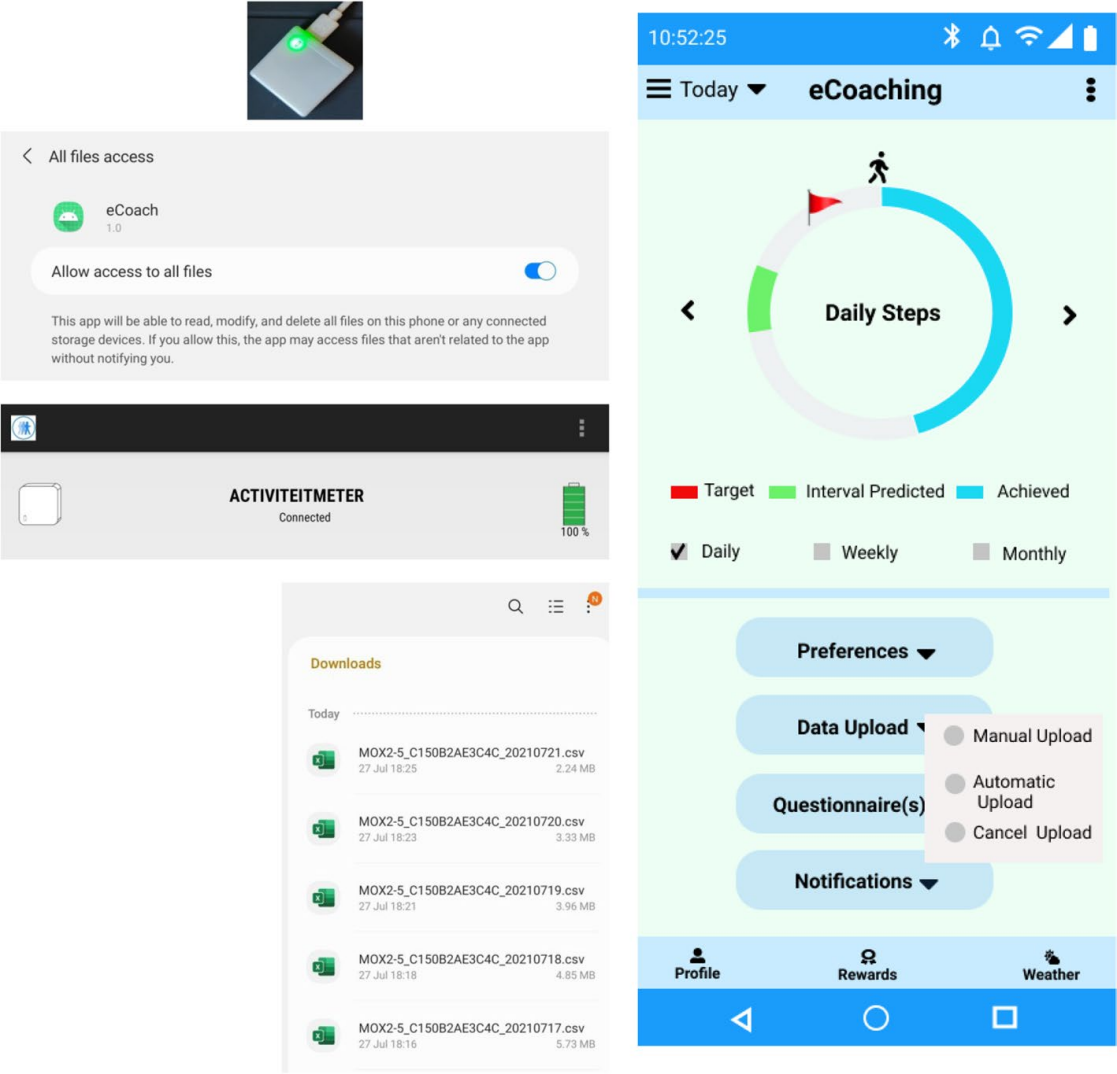
Sensor-based data collection interfaces in the eCoach prototype app

**Fig. 6:**
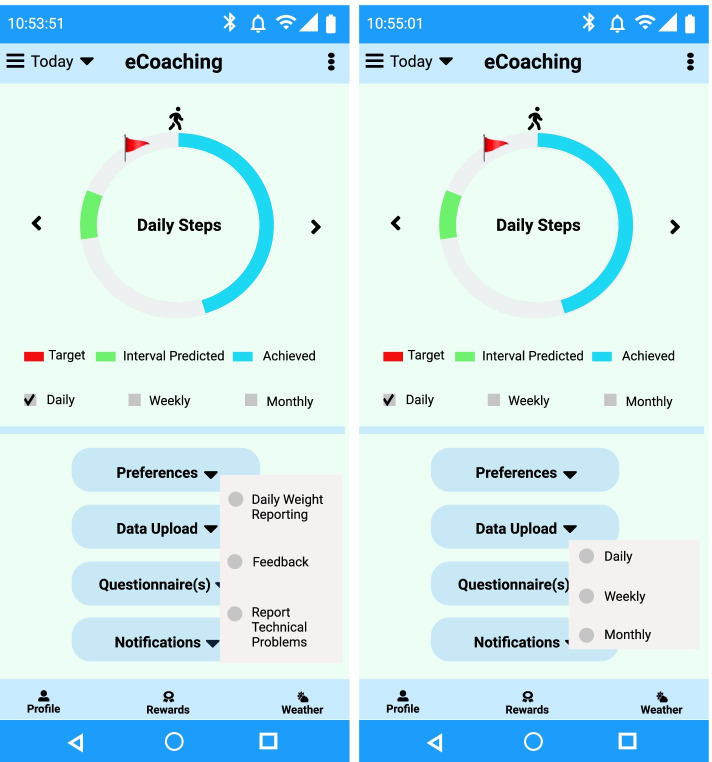
Options for questionnaire-based data collection and historic or current notification visualization interfaces in the eCoach prototype app

The MOX2–5 activity sensor is a 3-dimensional accelerometer with a 25–100 Hz sample rate (dimensions 35 × 35 × 10 mm). Its sensitivity is 4 mg/LSB. Maastricht Instruments had developed it. It is dust and waterproof gives a battery backup for 7 days, and is built with a rechargeable “Lithium Ion125 mAh”. The current version of the MOX2–5 activity sensor is not suitable for classifying activities into the following detailed activity classes: cycling, swimming, rowing, and skiing. Therefore, the participants must report them manually as questionnaire data in the latest version of the eCoach app. The MOX2 sensor-based and questionnaire data collection interfaces of the eCoach app are depicted in Figs. 5 and 6. The daily weight reporting data will help to analyze if the regular physical activities or behavioral motivations impact gradual weight change. It can be a helpful direction in obesity and overweight case study with eCoaching.

#### Preference settings

**Fig. 7:**
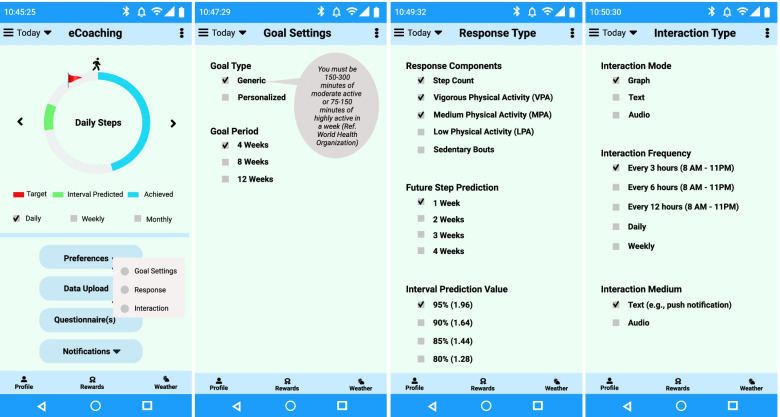
Representation of preference settings in the eCoach prototype app

We have designed interfaces for the questionnaires to collect personal preference data, such as goal setting, response, and interaction (see Fig. 7). There are two goal types – system-defined general goals for staying active following the guidelines of WHO and person-defined goals (as athletes might want to get coached towards specific training goals). The duration of the goal period can be 4–12 weeks or more based on personal preferences. The goal-setting can be short-term (e.g., daily, weekly) or long-term (e.g., bi-weekly, monthly). The eCoach system should encourage end-users to reach their long-term goals with the generation of tailored recommendations and the achievement of short term goals.

In our eCoach app, we have considered the following pre-selected default values for the preference settings and the graphical user interface (GUI) design are depicted in Fig. 7.

- Goal Type: Generic or Personalized
- Goal Period: 4 weeks
- Response Type: Representation of steps, VPA, MPA, LPA, sedentary bouts, future step prediction and interval prediction value
- Interaction Mode: Graph, Text, Audio
- Interaction Frequency: Regular interval, Daily, Weekly
- Interaction Medium: Text (e.g., push notification), Audio

**Fig. 8:**
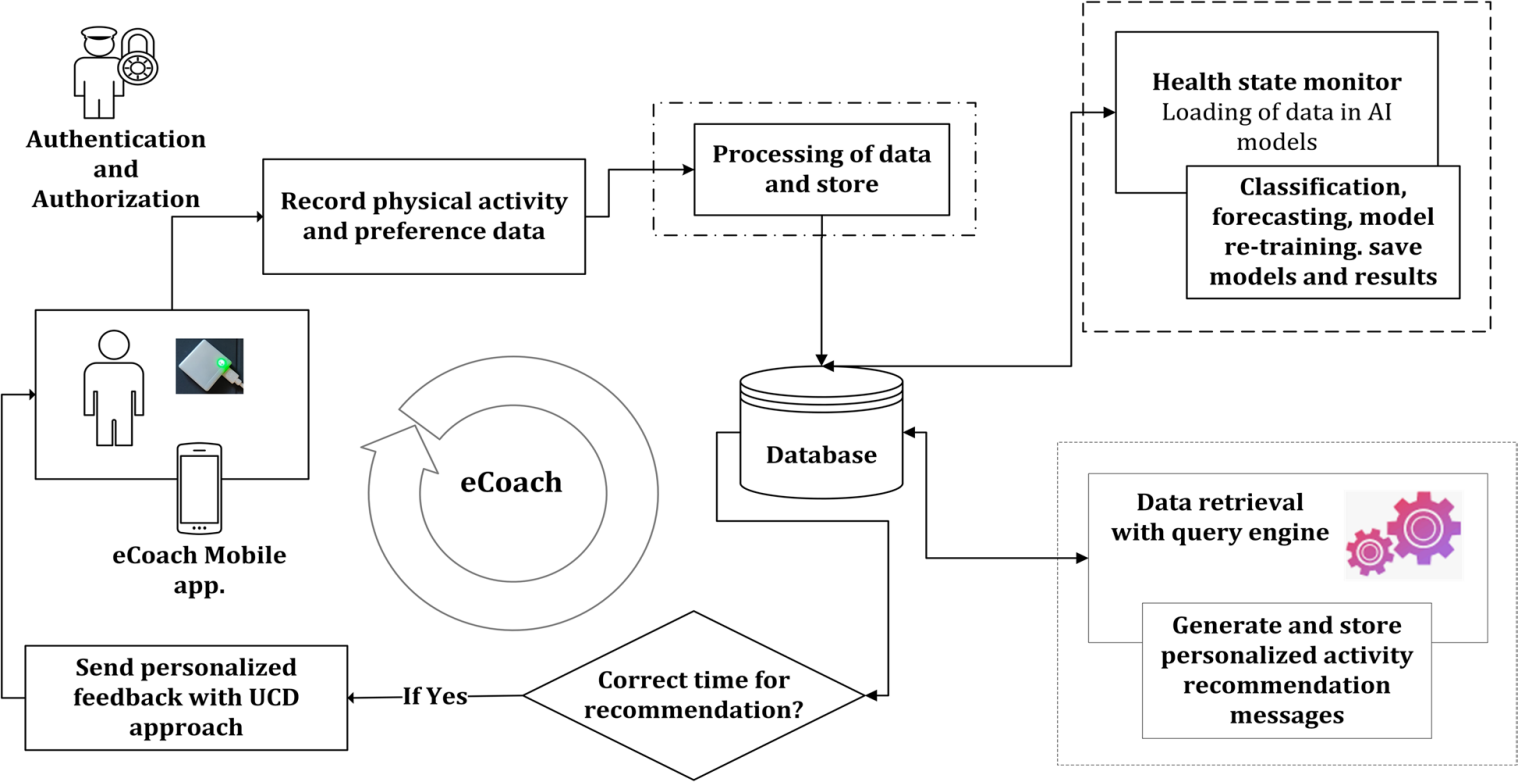
The continuous process of personalized data collection, decision making and personalized hybrid recommendation generation combining AI-results and query rules

All the preference and physical activity data are recorded in a relational database using semantic annotation. Individuals are always allowed to view and update their preference data. A hybrid (data and rule-driven) health state monitoring component is responsible for analyzing physical activity progress and followed by the generation of recommendations to reach personal activity goals (see Fig. 8).

#### Monitoring and recommendation (feedback) visualization

The app keeps track of an individual number of steps, duration of VPA, MPA, and LPA (in minutes per day), and sedentary bouts until the monitoring period gets over. Participants can actively monitor or track the number of exercises they have performed over the day or week based on their preferences. They will have the option to see their historical performances as well. At the end of the eCoaching session, they can report notes on their satisfaction with using the app. In UCD workshop 2, end-users showed interest in simplified metrics. Therefore, the eCoach app provides numerical feedback on the activity performed on simplified graphs. Here, feedbacks are of two types to motivate participants – indirect visual feedback and direct (e.g., textual pop-up notification generation). The participant receives daily as well as cumulative feedback at the end of the session to view their progress towards the goal.

In our activity eCoaching app, we have considered a hybrid health state monitoring component. During health state assessment, the module can predict the activity pattern of the participants (e.g., steps), automatically for the next “n” days (*n* > 0) based on the temporal pattern in data. It can help participants to identify which kind of activities they should perform to reach their long-term goals. Temporal analysis on data (e.g., deviation in activities) helps to analyze the pattern in human activities and generate evidence-based tailored recommendations to motivate participants (e.g., comparative statistical analysis in activity data between weeks W1, W2, and W3 helps to determine if any deviation or improvement in performance or in which week the participant was more active). These recommendations can be contextual with the inclusion of weather information (e.g., tomorrow morning, the weather is sunny, and temperature is between 15 and 18-degree Celcius (C). Therefore, you can plan to walk for 1 h or perform similar activities).

We have formatted activities in minutes per day or steps per day instead of calories which is inaccurate and difficult to understand for the users how calories relate to the activity goal. Moreover, for estimating future activity in terms of “steps” based on time-series monitoring data processing using deep learning-based forecasting, we focused on probabilistic interval prediction rather than abstract point prediction. A prediction interval gives an interval within which we expect to remain with a specified probability.

A prediction interval can be written as,

$${Y}_{T+h\mid T}\pm c\ast {\sigma}_h$$

Where, “c” depends on the coverage probability and in one-step interval prediction its value is 1.96 (95% prediction interval where forecast errors are normally distributed). "*σ*_*h*_^"^ is the estimation of the standard deviation in the h-step forecast distribution (h > 0). However, deep learning-based forecasting implementation, calculation of residual errors in temporal step data, and h-step prediction interval calculation is beyond the focus of this paper. By default, we have used c = 1.96. However, participants can choose the value of “c” up to 1.28 (80% interval).

**Fig. 9:**
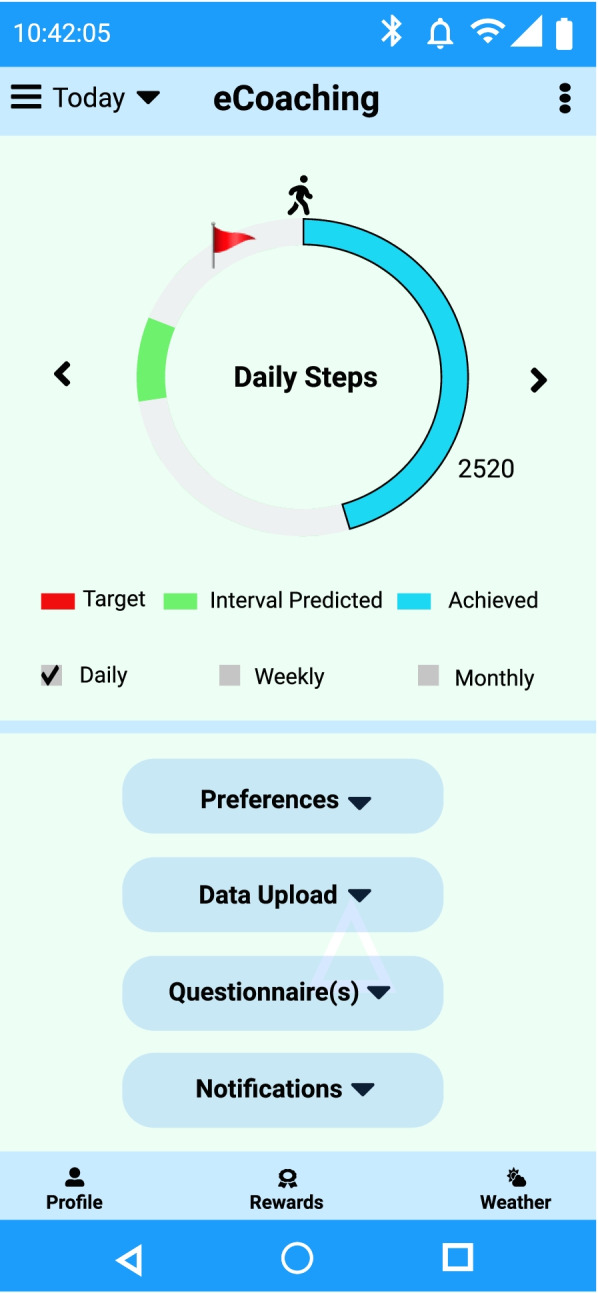
Visualization of daily step count, target step count and predicted interval

In UCD workshop 2, end-users agreed to visualize their activity intensity simplified and briefly. Therefore, we had not considered infantile animations, sound like feedback when goal achieved as they might cause unnecessary interruptions. We have prioritized weekly performance evaluation rather than daily performances as participants can be active and maybe less active on the next day. A balance of activities must be maintained to achieve the short-term weekly goals to reach long-term monthly goals. We have shown a sample recommendation visualization screen in Fig. 9. In the figure, daily step count has been represented with a target daily step count based on the goal settings deep learning-based step prediction, and its extension with naïve-based interval prediction. The nature of step prediction is dynamic and depends on the steps achieved.

#### Notification

**Fig. 10:**
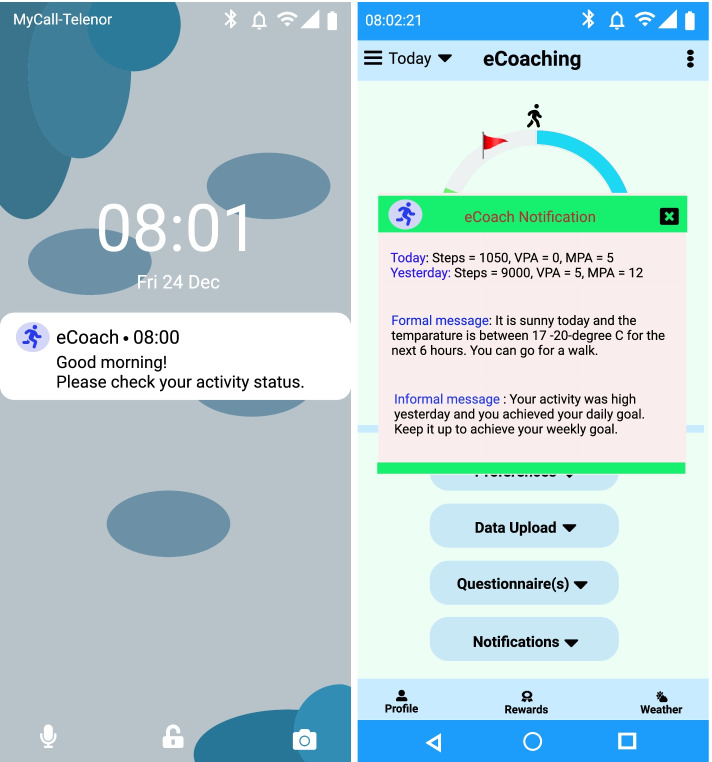
A sample notification at 8 am. and its visualization in the eCoach app

The recommendation module generates personalized and contextual recommendations based on the predicted health state. Recommendations can be direct (for example, pop-up notifications or alerts) or indirect (for example, activity status visualization). Instant notifications can contain two types of messages: (a.) formal To-Do (for instance, “You need to complete another 1500 steps in the next three hours to reach your daily goal”) and (b.) informal motivational notifications (e.g., “Good job, Keep going! You have achieved targeted steps.”). In the activity eCoaching framework, the messages are annotated in a semantic ontology. To inform the user about activity in progress, we have used the indirect approach for recommendation visualization, and to give direct instant notifications, we have considered pop-up text alerts. The participants can select the notification frequency as part of the app preferences. By default, we have considered activity notifications every 3 h between 8 AM and 11 PM; however, the user can modify that. These notifications are timely alerts. It will help participants to stay on the right track either with motivational messages or with activity improvement suggestions. Notifications have been kept short, understandable, and positive. We have depicted sample push notification generation screens in Fig. 10.

#### Rewards

**Fig. 11:**
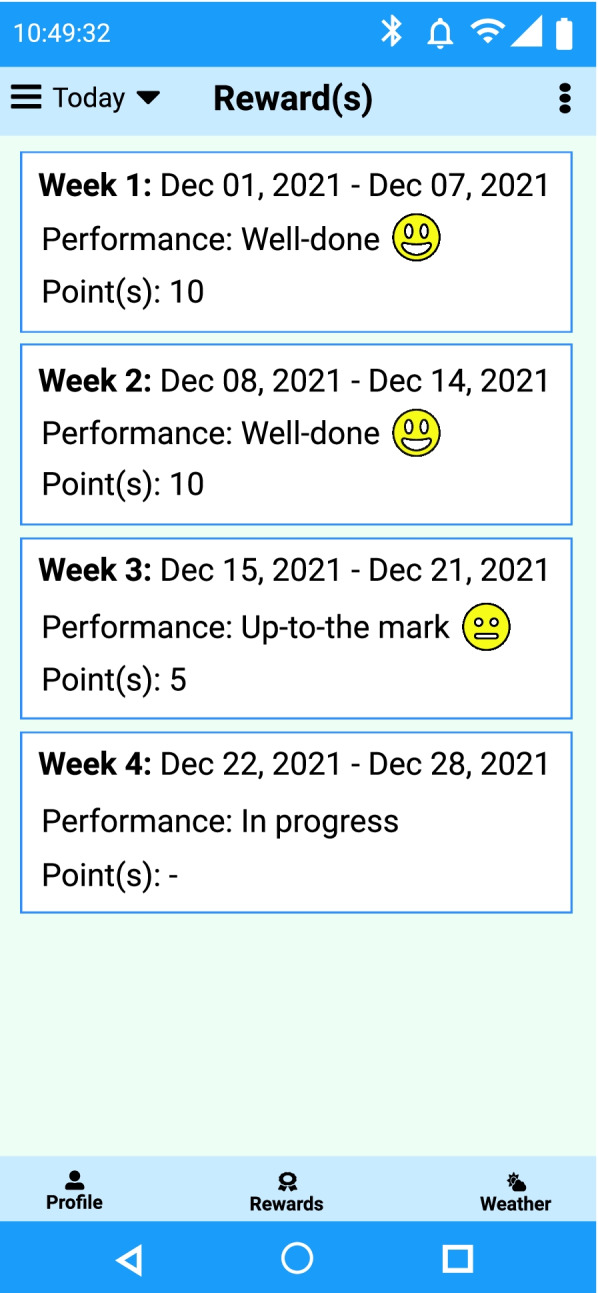
An example weekly reward generation screen in the eCoach app

We have considered a simple emoji and a textual message to represent individuals’ short-term (e.g., daily weekly) and long-term (e.g., bi-weekly, monthly) goals. We have used three emojis to classify individual progress to reach personalized goal into three groups – well done (  ) [10 credit points], up-to-the mark (  ) [5 credit points], must be improved (  ) [0 credit point]. All the credit points can be reimbursed against “Food bank”, as “reward” means that the user can eat a bit more, if he has trained more. We will decide to offer a list of potential food items in the “Food bank” against weekly accumulated credit achieved. It is a motivation to do more activity. In the future, we will enhance the reward generation with demographic clustering and profile ranking methods to motivate participants. We have depicted a sample personalized weekly reward generation in Fig. 11.

## Discussion

### Principal findings, innovation, and technology readiness

eCoach features [9] (such as recommendation, personalization, interaction, co-creation, goal management, automation, and persuasion) utilize a combination of wearable activity sensors and digital activity trackers with improving physical activity. An intelligent eCoach system can generate automatic, meaningful, evidence-based, and personalized lifestyle recommendations to achieve personal lifestyle goals. Real-time analysis of data to create customized recommendations on time is crucial in eCoaching. From the literature search point of view, the concept of eCoach in the healthcare field is still in its infancy. The associated studies can be classified into the following two categories: “What to coach” and “How to coach” In fact, in the original eCoach concept, data collection and processing is used to determine the “WHAT to coach”, in terms of the content of recommendations (direct or indirect), including the calculation of predicted activity, and the resulting gap to the set goal. The “HOW to coach” addresses the HCI related to turning human coaching into automatic, digital coaching, including aspects of persuasive technologies and motivational messages. Integration of recommendation technology with machine learning algorithms and its visualization in an appropriate way to motivate participants is another challenging task in an eCoach design and its development, and this has been addressed in this paper.

This study presents a detailed overview of rationale, characteristics, user-centered design, and the development process of a health eCoach app for the self-management of physical activity to reduce sedentary behavior or to stay active. Proper utilization of the activity eCoaching concept with positive psychology may open a direction for self-management of weight. Our intended eCoach app aims to increase individual participants’ apparent abilities and motivation with monitoring and feedback generation and to trigger participants to engage in physical activities at the right time by leveraging self-maintained persuasive strategies. In the design and development of this app, the research team collected user needs and preferences, and an engineering team interpreted them into technical requirements, specifications, and technical solutions. This app allows changing behavior and habits by increased self-knowledge, self-monitoring, self-awareness, and self-effectiveness. Personalized preferences are set, and tailored evidence-based contextual feedbacks are generated based on the degree of goal achievement. The UCD approach provided an understanding of the needs of end-users to make the design of our eCoach app successful. The main requirements for the app design and development as derived from the UCD approach were:

- Data sharing must conform to the GDPR regulations and ethical guidelines [41, 42].
- Data comes from heterogenous sources. There must be a method to annotate data.
- Selection of appropriate medical grade activity sensor that can measure activity accurately. Data collection should not create additional burden to the participants. Proper placement of the sensor so that it does not create any nuisance.
- Settings of preferences based on individual needs.
- Feedback or recommendations must be direct or indirect. Recommendations should be personalized, evidence-based, contextual, periodic, comprehensible, subtle, brief, and simplified.
- Simple rewarding mechanism to motivate participants.

We integrated the Semantic Sensor Network (SSN) ontology and selected concepts from Systematized Nomenclature of Medicine - Clinical Terms (SNOMED-CT) into our ontology model used in the eCoach Framework to annotate data [3, 43–45]. However, the ontology design and its implementation are beyond the scope of this paper. In the open discussions (design workshops), end-user groups were not agreed on all requirements. Questions raised for using the MOX-2 device for activity monitoring as there are different activity monitoring mechanisms in the market, such as Apple, Samsung, and other consumer devices (e.g., Fitbit, Actigraph, smart-watches). Maastricht Instruments, a spin-off company of the Maastricht Hospital, and supplier of the MOX-2 activity monitoring device, has informed about the following:

- Apple, Samsung or similar service providers utilizes the sensors in the smartphone. People do not wear the smartphone at the same body location all around the day, so this poses difficulty in accurately assessing physical activity. On the other side, a higher-level activity information is possible, such as including location information from the smartphone.
- Maastricht Instruments validated Fitbit, and the like in elderly populations, and they saw a high variability. Furthermore, it is never known when the manufacturer replaces the algorithms or sensors in the device, so it is tricky to do clinical trials with such devices (over longer durations).
- Most consumer devices are not suitable for use in medical applications. Maastricht Instruments has prove the performance of their MOX-2 device in several published studies on elderly and disease populations [18].
- Actigraph is in the same category of devices as the MOX; however, MOX2–5 is cheaper to use for clinical trials.

Our concept of eCoaching is novel and in contrast to prove the hypothesis that - “in eCoaching, automatic generation of personalized recommendation is possible”. Here, we collected design requirements from the end-users to develop an app that can generate effective individualized recommendation in a sedentary lifestyle and turn it into a behavioral motivation for an effective human-eCoach-interaction. In the eCoach system, the concept of transforming distributed, heterogenous health and wellness data (e.g., sensor, questionnaire) into meaningful information with semantic ontology is inventive. Here, we used AI-inspired recommendation technology, processing of medical-grade sensor data, anomaly detection in data and its removal, residual error minimization to improve the time-series prediction, and probabilistic interval prediction rather abstract point prediction for motivational recommendation visualization to make the solution pioneering. Moreover, the adoption of persuasive strategies in the app design has made the concept innovative. In.

Table 5, we have performed a qualitative comparison between our ProHealth eCoach and commercial activity tracking smartphone apps (e.g., Fitbit, Actigraph, MOX2–5, Pedometer, Garmin, and smartwatches (e.g., Apple, Samsung, Huawei)) regarding eCoach components identified in the literature search [5, 9]. Traditional activity tracking smartphone apps focus more on data capturing and its representation; however, they suffer from UCD approach, adequate data, data protection, data consistency, proper documentation, guidelines, and ethical approvals. Table 6 describes technological readiness levels (TRLs) of ProHealth eCoach against standard levels set by EU [46, 47].

**Table 5:** A qualitative comparison in regarding to the generic eCoaching components

Persuasive eCoaching components	Addressed in commercial activity tracking mobile apps including smartwatches?	Addressed in ProHealth eCoach?
Intervention	No	Yes
Personalization	No	Yes
Interaction	Yes	Yes
Co-creation	No	Yes
Goal-settings and evaluation	No	Yes
Automation	No	Yes
Persuasion	No	Yes
Goal-based personalized recommendation generation	No	Yes

**Table 6:** Achieved TRLs by our ProHealth eCoach

Number(s)	Technology readiness levels	Achieved (Yes/No)?	Comment(s)
TL-1	Basic principles observed	Yes	–
TL-2	Technology concept formulated	Yes	–
TL-3	Experimental proof of concept	Yes	–
TL-4	Technology validated in lab	Yes	–
TL-5	Technology validated in relevant environment (industrially relevant environment in the case of key enabling technologies)	No	We will evaluate this in our future usability study.
TL-6	Technology demonstrated in relevant environment (industrially relevant environment in the case of key enabling technologies)	No	We will evaluate this in our future usability study.
TL-7	System prototype demonstration in operational environment	No	We have designed and developed an initial version of the eCoach prototype; however, integration and scalability testing must be performed in the production environment.
TL-8	System complete and qualified	No	Usability evaluation must be performed on a group of participants for further model improvement and qualification.
TL-9	Actual system proven in operational environment (competitive manufacturing in the case of key enabling technologies; or in space)	No	The system will be operational after efficacy evaluation of the eCoach app. on a group of controlled trials.

### Limitations and future scope

We plan to overcome certain limitations of this study in our future work. The restrictions are summarized as follows – First, we have presented the design and development of an eCoach prototype (i.e., ProHealth eCoach) for activity coaching. However, we have not performed its usability testing for the heuristic evaluation of the eCoach prototype. Second, in activity monitoring, the scope can be extended to sleep monitoring rather than only step prediction and visualization along with daily step count and total minutes of VPA, MPA, and sedentary bouts. Third, this study has not evaluated recommendation generation’s credibility, reliability, and effectiveness and its presentation (direct and indirect) towards motivational, behavioral change. Following usability evaluation, we will recruit participants of similar interests for efficacy evaluation of the recommendation generation. Fourth, constraints, such as poor internet connectivity, battery lifetime due to BLE and background processing, budget, time plan, technological limitations should be overcome. Fifth, the sensor cannot distinguish the type of activities, such as swimming, skiing, cycling. Therefore, a questionnaire should be designed to overcome it’s reporting. Sixth, the scope of recommendation generation and turning it into a behavioral motivation is extensive. Here, we have not evaluated concepts, such as what is a good goal? How to generate effective feedback for behavioral motivation? Future studies can compare actual participants’ feedback and activity trends to modify goal settings and gradually tailor them. Likewise, recommendations can be presented to participants in different ways, such as visual (e.g., graph, chart), audio, text (e.g., pop-up notification or on-screen messages), or any combination. In our future study, we can recruit different people to compare the conceptual basis of effective recommendation presentation for behavioral motivation. Seventh, besides only activity monitoring and recommendation generation, incorporation of nutrition assessment and the tracking of habit can allow eCoach app to change behavior for a healthy lifestyle in obesity case. Eighth, improvement in AI prediction to classifiy between meaningful (effective) and bad (inefficient) recommendations with a process of continuous learning from individual data and performance trends, and following, personalized recommendation generation with obtained knowledge. Ninth, here we have discussed eCoaching for personalized physical activity monitoring with tailored recommendation generation, self-monitoring, motivation, and goal management. However, eCoaching can be broad in controlling other behavioral changes, such as habit, nutrition, depression, chronic pain, and cognitive decline. Therefore, the eCoaching concept can be promising in preventing chronic illnesses, such as diabetes type II, obesity and overweight, mental health, and cardiovascular rehabilitation. Tenth, recommendations in an eCoach system can be rule-based, data-driven, or hybrid. An appropriate selection of recommendation generation methods is essential in eCoaching to generate contextual and meaningful personalized and group-level recommendations. Adoption of explanation methods in recommendation generation will make eCoaching more attractive and trustworthy to its participants. Eleventh, behavior is a slow but gradual change. To evaluate the practical efficacy of eCoaching toward behavior change, self-management, credibility, and motivation, a proper longitudinal study plan is necessary for two groups (one group without eCoaching and one group with eCoaching) of controlled trials with a minimum group size of 50 participants following inclusion and exclusion criteria, to compare the outcomes with statistical methods. Furthermore, future work focuses to understand the importance of socio-demographic characteristics such as age, gender, ethnicity, education level etc. of the enrolled individuals to achieve a high level of generalised findings. It also helps to categories individuals into different sub-groups to obtain effective support to control their lifestyle and behaviors for more generalised purposes.

## Conclusions

In this study, the design and implementation process of an activity eCoach monitoring and personalized recommendation generation app is described as the preparation of a mHealth intermediation to encourage the self-management of PA. It demonstrates a user-centered design process’s consideration to make it suitable for end-user, technology, healthcare professionals, engineers, and researchers. The main principle of this eCoach app is to change an individual’s sedentary behavior by self-monitoring, preference setting, personalized recommendation generation, and its presentation. The app connects three technologies – an accelerometer-based medical-grade activity sensor, an android mobile app, and an internet application. The eCoach app design directs to an innovative approach with the adoption of the following concepts – persuasive strategies, ontology-based data annotation, hybrid recommendation technology, interval prediction, and the incorporation of medical-grade activity sensors. Following the user-centered design, the usability and efficacy evaluation of the eCoach app will be engaged in the lab environment and a cluster of a controlled trial, respectively.

## Supplementary Information

**Additional file 1.** StaRI checklist for completion.

**Additional file 2.** The outcome of the focus group discussion for RQ-1 in Workshop 1.

**Additional file 3.** The outcome of the focus group discussion for RQ-2 in Workshop 1.

**Additional file 4.** The outcome of the focus group discussion for RQ-3 in Workshop 1.

**Additional file 5.** The outcome of the focus group discussion for RQ-4 in Workshop 1.

**Additional file 6.** Here, we have attached a video demonstration of the activity eCoach app’s a high-level working as a supplementary ProHealth eCoach.mp4 file (2.46 minutes or min). The navigation windows are mentiond as follows: • Feature navigation (0.04 seconds or sec). ○ Login (0.09 sec). ○ Visualize step count (0.19 sec). ○ Navigate preferences (0.26 sec). ○ Navigate data upload (1.06 min). ○ Navigate questionnaire(s) (1.15 min). ○ Navigate notifications (1.24 min). • Notification and reward visualization (1.36 min). • Data collection with MOX2–5 activity sensor (1.55 min).

## Data Availability

All data generated or analysed during this study are included in this published article and its supplementary information files.

## Abbreviations

PA: Physical Activity
eCoach: Electronic Coach
mHealth: Mobile Health
UCD: User Centered Design
CVDs: Cardiovascular Diseases
WHO: World Health Organization
IDEAS: Integrate, Design, Assess, and Share
MRC: Medical Research Council
BIT: Behavioral Intervention Technology
COM-B: Capability, Opportunity, Motivation, and Behavior
FBM: Fogg’s Behavior Model
JITAI: Just-In-Time (Adaptive) Interventions
HCI: Human-Computer-Interaction
IoT: Internet-of-Things
USA: United States of Amderica
FDA: Food and Drug Administration
RQ: Research Questions
GDM: Gestational Diabetes Mellitus
WIISEL: Wireless Insole for Independent and Safe Elderly Living
NSD: Norwegian Centre for Research Data
AI: Artificial Intelligence
REK: Regional Committees for Medical and Health Research Ethics
SMART: Specific, Measurable, Attainable, Relevant, and Time Bound
LPA: Low Physical Activity
MPA: Medium Physical Activity
VPA: Vigorous Physical Activity
UUID: Unique User Identifier
BLE: Bluetooth Enabled
REST: Representational State Transfer
API: Application Programming Interface
GDPR: General Data Protection Regulation
NORMEN: Norm for information security and privacy in health
GUI: Graphical User Interface
SSN: Semantic Sensor Network
SNOMED-CT: Systematized Nomenclature of Medicine - Clinical Terms
BMI: Body Mass Index
